# Poly(Ethylene Glycol) Crosslinked Multi-Armed Poly(l-Lysine) with Encapsulating Capacity and Antimicrobial Activity for the Potential Treatment of Infection-Involved Multifactorial Diseases

**DOI:** 10.3390/pharmaceutics12010047

**Published:** 2020-01-06

**Authors:** Chao Lu, Ting Wen, Maochao Zheng, Daojun Liu, Guilan Quan, Xin Pan, Chuanbin Wu

**Affiliations:** 1 School of Pharmaceutical Sciences, Sun Yat-Sen University, Guangzhou 510006, China; 2 College of Pharmacy, Jinan University, Guangzhou 510632, China; 3 Department of chemistry, Shantou University Medical College, Shantou 515041, China

**Keywords:** polyion complex micelles, shell crosslinked micelles, drug delivery systems, antimicrobial polymer, multifactorial diseases

## Abstract

With the development of modern medical technology, common diseases usually can be treated by traditional medicines and their formulation, while diseases with multiple etiologies still remain a great challenge in clinic. Nanoformulation was widely explored to address this problem. However, due to limited drug loading space of nanocarriers, co-delivery strategy usually fails to achieve sufficient loading of multiple drugs simultaneously. In this research, we explored the potential of poly(ethylene glycol) (PEG) crosslinked alternating copolymers MPLL-*alt*-PEG as both an anionic drug carrier and antimicrobial agent. The high cationic charge density of multi-armed poly(l-lysine) (MPLL) segments in MPLL-*alt*-PEG could endow the electrostatic encapsulation of anionic model drugs through the formation of polyion complex micelles with a MPLL/drug complex core and crosslinked PEG outer shell, enabling pH-sensitive drug release. Meanwhile, the MPLL-*alt*-PEG copolymer exhibits a broad spectrum of antimicrobial activities against various clinically relevant microorganisms with low hemolytic activity. Studies on antibacterial mechanism revealed that MPLL-*alt*-PEG attacked bacteria through the membrane disruption mechanism which is similar to that of typical antimicrobial peptides. Taken together, the present study shed light on the possibility of endowing a polymeric carrier with therapeutic effect and thus offered a promising strategy for achieving a comprehensive treatment of bacterial infection-involved multifactorial diseases.

## 1. Introduction

In the past decades, nanotechnology has been well developed to construct drug delivery systems, including but not limited to micelles, liposomes, and nanoparticles [1,2,3,4,5,6,7,8,9,10]. Compared with conventional formulations, these nanoscaled drug delivery systems (DDS) exhibit great potential in improving drug stability, preventing premature drug release, altering drug distribution, and prolonging the half-life of drugs [11]. Therefore, they are widely applied in the delivery of a great variety of drugs including anticancer drugs [1,2], antimicrobial agents [3,4], and anti-inflammatory drugs [5,6], among others.

Though showing unique advantages in delivering drugs for monotherapy, the nanoscaled DDS still suffer from many limitations in enhancing the comprehensive treatment efficacy of disease with multiple etiologies. In fact, many common clinical diseases are caused or worsened by the interplay of multiple factors. For example, burn wound infection is often aggravated by the infection of bacteria, such as *Pseudomonas aeruginosa* (*P. aeruginosa*) and *Staphylococcus aureus* (*S. aureus*) [12]. When a massive loss of liver parenchyma and metabolic function induces acute liver failure, patients will become susceptible to pathogens, which largely limits the feasibility of advanced emergency liver transplantation [13]. It was also reported that bacterial infection might be one of the main reasons to induce tumour formation, so both anticancer and antibacterial measures should be employed for the treatment of these diseases, such as *Helicobacter pylori*-induced gastric carcinoma [14]. Therefore, to treat multifactorial diseases, it is critical that DDS should be rationally developed to co-deliver therapeutic agents and antimicrobial agents such as antibiotics [3], antimicrobial peptides [15,16], metal ions [4], and natural compounds [17].

To achieve this, one of the most common strategies is to co-deliver two or more drugs in one formulation [18,19]. However, due to limited drug loading space of nanocarriers, the co-delivery strategy usually fails to achieve sufficient loading of multiple drugs simultaneously [18,19,20]. Recently, poly(l-lysine) (PLL)-based polymers have been fabricated to encapsulate drugs with a negative charge, such as small molecule drugs [21,22,23], proteins [24,25], and nucleic acids [26,27]. Notably, Qiao et al. [15] found that PLL-based polymers exerted a bactericidal mechanism similar to that of common antimicrobial peptides (AMPs), possessing a great potential to eradicate bacterial infections. Our previous research [16] also demonstrated that multi-armed PLL (MPLL), with high antimicrobial activity, enormous selectivity, and remarkable proteolytic stability, represented a new series of potent antimicrobial peptides to treat drug-resistant infections [16]. All these research advancements prognosticated that PLL-based polymers could be served not only as drug carriers but also as macromolecules with therapeutic activity to synergize therapeutic effect, which implied a new perspective for the design of nanomedicine against multifactorial diseases.

**Scheme 1:**
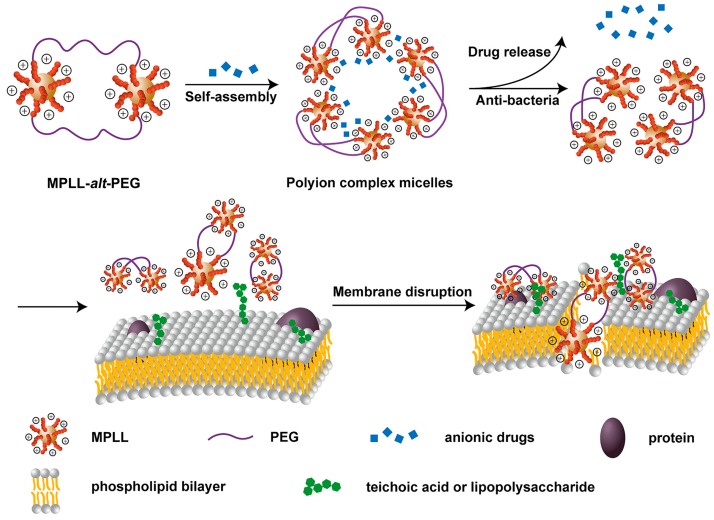
The schematic formation of drug-loaded polyion complex (PIC) micelles and their mechanism of action against bacteria through membrane disruption. MPLL: multi-armed poly(l-lysine); PEG: poly(ethylene glycol).

In this research, poly (ethylene glycol) (PEG) crosslinked alternating copolymers in the form of MPLL-*alt*-PEG (Scheme 1) were designed, synthesized, and evaluated as a platform for both anionic drug delivery and anti-bacteria. MPLL with a branched polyethylenimine (PEI) core and poly(l-lysine) peripheral chains comprised the polyelectrolyte segment of MPLL-*alt*-PEG copolymer. Their multi-armed molecular architecture possessed high cationic charging density, which is beneficial for enhancing the binding affinity towards anionic drugs and bacterial lipid bilayers via electrostatic interactions [16]. It is expected that MPLL-*alt*-PEG could encapsulate anionic drugs, followed by self-assembly into shell crosslinked polyion complex (PIC) micelles (Scheme 1). Our previous research suggested that shell crosslinked micelles could possess unique advantages in effective drug loading, improving micellar stability, and protecting the drug from premature release and degradation [28]. Meanwhile, the antimicrobial activity of MPLL could also be improved after crosslinking since the crosslinking reaction would lead to increased molecular weight, proteolytic stability, and in vivo half-life [16,29]. The drug-loaded MPLL-*alt*-PEG micelles could integrate the therapeutic effect of loaded drugs and the antimicrobial activity of carriers. We demonstrated the feasibility of MPLL-*alt*-PEG to serve as a DDS for small molecule drugs and therapeutic biomacromolecules, using water-soluble methyl orange (MO) and immunotropic agent recombinant human interferon α-2b (IFN) as anionic model drugs. Subsequently, the antimicrobial activity and mechanism of MPLL-*alt*-PEG was investigated using Gram-positive methicillin-resistant *Staphylococcus aureus* (MRSA) and Gram-negative *P. aeruginosa* as model bacteria. We expected that the MPLL-*alt*-PEG-based nanoplatform could be applied as a potential system to treat bacterial infection-involved multifactorial diseases.

## 2. Materials and Methods

### 2.1. Materials

Branched PEI (Mw = 0.8 kDa) were obtained from Sigma-Aldrich (Shanghai, China). ε-Benzyloxycarbonyl-l-lysine (ZLL) was purchased from GL Biochem (Shanghai, China) and used without further purification. PEG (succinimidyl carboxymethyl ester)_2_ (SCM-PEG-SCM, Mw = 7.5 kDa) was supplied by JenKem Technology Co., Ltd. (Beijing, China). Methyl orange, anisole, methanesulfonic acid, trifluoroacetic acid (TFA), and fluorescein isothiocyanate (FITC) were all obtained from Aladdin (Shanghai, China). A dialysis tube with molecular weight cut-off (MWCO) values of 7 and 14 kDa was produced by Viskase (Darien, IL, USA), while dialysis tubes of 50 kDa and 100 kDa were purchased from Spectrum Laboratories Inc. (Los Angeles, CA, USA). IFN (5 million U) was obtained from Anhui Anke Biotechnology Co., Ltd. (Anhui, China).

### 2.2. Synthesis and Characterization of MPLL-alt-PEG Copolymers

**Figure 1:**
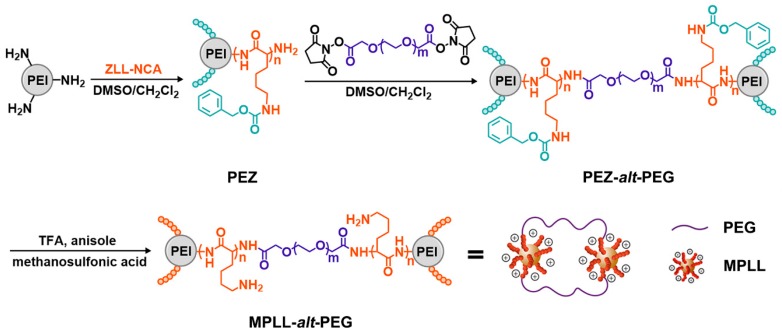
The synthesis route of MPLL-*alt*-PEG copolymers. PEI: polyethylenimine; PEZ: PEI-grafted poly(ε-benzyloxycarbonyl-l-lysine); TFA: trifluoroacetic acid.

MPLL-*alt*-PEG copolymers were synthesized in a successive procedure of PEI initiated polymerization of ε-benzyloxycarbonyl-l-lysine *N*-carboxyanhydride (ZLL-NCA) to prepare PEI-grafted poly(ε-benzyloxycarbonyl-l-lysine) (PEZ), followed by the peripheral crosslinking with difunctional SCM-PEG-SCM, and the subsequent removal of the benzyloxycarbonyl side chain protecting groups (Figure 1).

In the first step, ZLL-NCA was prepared via phosgenation of ZLL in anhydrous ethyl acetate (Yield: 78%) and then polymerized using PEI as the macroinitiator to obtain the star-block copolymers PEZ (Yield: 92%) according to our previous report [16,24,28,30].

Then, 100 mg of PEZ was dissolved in a mixture of 50 mL of DMSO and 250 mL of dichloromethane (DCM). The solution of SCM-PEG-SCM at a concentration of 0.2 g·mL^−1^ in DCM was added dropwise into PEZ solution under vigorous stirring conditions at 25 °C for 24 h to produce PEZ-*alt*-PEG [28]. The solution was concentrated by rotary evaporation to remove DCM and dialyzed (MWCO 14 kDa) against water. Subsequently, PEZ-*alt*-PEG copolymers were obtained by lyophilization. Yield: 95%.

Finally, PEZ-*alt*-PEG was dissolved in TFA, followed by the addition of anisole and methanesulfonic acid. After stirring at room temperature for 1.5 h, the solution was diluted with distilled water and washed three times with diethyl ether. The aqueous layer was collected and then neutralized with sodium bicarbonate. Afterwards, the sample was transferred into a dialysis tube (MWCO 7 kDa) and dialyzed for two days against a 5.0 wt% NaHCO_3_ aqueous solution and subsequently five days against distilled water. The dialyzed product was then lyophilized to obtain the final product MPLL-*alt*-PEG. Yield: 91%

The ^1^H nuclear magnetic resonance (NMR) spectra of PEZ and PEZ-*alt*-PEG were determined on a Bruker DMX 400 MHz spectrometer (Bruker, Karlsruhe, Germany) using DMSO-*d*_6_ as solvent, while MPLL-*alt*-PEG was characterized with D_2_O as solvent. The molecular weights of various copolymers were determined by gel permeation chromatography (GPC) systems (Waters, Milford, USA) equipped with a Waters 515 HPLC pump and a Waters 2414 refractive index detector. For the characterization of PEZ and PEZ-*alt*-PEG, samples were analyzed with dimethylformamide (DMF) as the eluent using linear poly(methyl methacrylates) (PMMAs) as standards. For the characterization of MPLL-*alt*-PEG, the sample was measured using a 0.50 M HAc–NaAc buffer (pH 4.5) as the eluent and linear PEG as standards.

### 2.3. Preparation and Characterization of Drug-Loaded MPLL-alt-PEG Micelles

To evaluate the encapsulation of small anionic molecules by MPLL-*alt*-PEG, a given amount of MO was added into a MPLL-*alt*-PEG solution in water (10 mL) and stirred for 5 min. The obtained solution was then dialyzed (MWCO 50 kDa) against water under sink conditions to remove free guest molecules. The solution with an equivalent amount of free MO was selected as the control for dialysis under the same conditions. When the MO solution in the control group was dialyzed to colorless, MO/MPLL-*alt*-PEG micelles were obtained, and the amounts of MO in the dialysate was determined by UV–vis spectrophotometer. A standard calibration curve was used for quantification of MO with linear correlation coefficient (*r*^2^) > 0.99 in the concentration range of 0.1–6 mg·L^−1^, and the drug loading capacity was calculated as the mass percentage of MO to polymer.

To evaluate the encapsulation of anionic biomacromolecules by MPLL-*alt*-PEG, IFN was labelled by FITC following the reported method [31]. Subsequently, the MPLL-*alt*-PEG and FITC-labelled IFN (FITC-IFN) solution were dissolved in a 0.01 M phosphate buffer (pH 7.4) at a concentration of 2 mg·mL^−1^, respectively. 1 mL of the IFN solution was then added dropwise into 5 mL of polymer solution under sonication for 5 min on a sonicator (KQ-200 KDE, Kunshan Ultrasonic Instrument Co., Kunshan, China, 40 kHz, 100 W), and then dialyzed (MWCO 100 kDa) against 0.01 M phosphate buffer (pH 7.4) under sink condition. The dialysis of free FITC-IFN in a 0.01 M phosphate buffer (pH 7.4) was also conducted as a control. When the free IFN in the control experiment had been completely removed, the amount of FITC-IFN in the outer dialysate was measured by a Spectramax Gemini XS microplate fluorometer (Molecular Devices Cooperation, Sunnyvale, CA, USA) using a standard calibration curve of FITC-IFN, and then that value was subtracted from the total amount of added FITC-IFN to calculate the loaded amount of FITC-IFN. The drug loading capacity was calculated as the mass percentage of FITC-IFN to polymer.

The morphologies of formed micelles were visualized with a JEOL JEM-1400 transmission electron microscope at an operating voltage of 100 kV (JEOL, Tokyo, Japan). The particle sizes and zeta potentials of both polymers and micelles were recorded at 25 °C using a Zetasizer Nano ZS90 (Malvern Instruments Ltd., Worcestershire, UK). The solutions were diluted to 1 mg·mL^−1^ using a 0.01 M phosphate buffer (pH 7.4) before testing.

### 2.4. pH-Sensitive Release of IFN from Micelle

FITC-IFN-loaded micelle solution (5 mL) was transferred into a dialysis bag (MWCO 100 kDa), and then the bag was placed into 150 mL of a 0.02 M phosphate buffer (pH 7.4), 0.02 M HAc–NaAc buffer (pH 5.5), or 0.02 M HAc–NaAc buffer (pH 4.5). The polymer/FITC-IFN complex solution was prepared as described in Section 2.3. At predetermined time intervals, a given volume of release media was withdrawn and replenished with an equal volume of fresh buffer solution. The amounts of released IFN were determined by the microplate fluorometer, the cumulative amount of released drug was calculated, and the percentages of drug released were plotted against time. All of the experiments were carried out in duplicate.

### 2.5. Measurement of Minimal Inhibitory Concentration (MIC)

The antimicrobial activities of the polymers were investigated against Gram-positive MRSA and Gram-negative *P. aeruginosa* using the broth microdilution method. Bacterial cells were incubated in a Mueller Hinton Broth (MHB) medium at 37 °C under constant shaking at 300 rpm to reach mid-logarithmic growth phase. The microbial suspensions were diluted and adjusted to 2 × 10^5^ colony forming units (CFU)·mL^−1^. Polymers were subjected to a series of two-fold dilutions using MHB to bacteria concentrations ranging from 60 to 0.2 μM. A total of 50 μL of each diluted polymer solution was added to 96-well microplates, followed by the addition of 50 μL of bacterial suspensions, to give a final inoculum of 1 × 10^5^ CFU·mL^−1^. After being incubated at 37 °C for 18 h, the optical density absorbance at 600 nm was measured with a microplate reader (Synergy HT, BioTek Instruments Inc., Winooski, VT, USA). Bacterial cells without polymer treatment and polymer solutions without bacteria addition were used as positive and negative controls, respectively. The MIC was defined as the lowest concentration that inhibited bacterial growth after 18 h.

### 2.6. Hemolysis Assay

Freshly mouse blood was treated with K_2_EDTA, washed with a phosphate buffer solution (PBS) three times and then centrifuged at 1000 *g* for 10 min to separate erythrocytes. Then, the red blood cells were obtained and diluted to a final concentration of 5% (*v*/*v*) with PBS (pH 7.4). 50 μL of polymer solutions at different concentrations were mixed with equal volume of red blood cell suspensions on a 96-well microplate followed by incubation for 1 h at 37 °C. After centrifugation at 1000× *g* for 10 min, aliquots (30 μL) of the supernatant were transferred to another 96-well microplate and diluted with 100 μL of PBS.

The release of hemoglobin from erythrocyte suspensions was monitored by absorbance measurement at 540 nm using a Biotek Synergy TH microplate reader. Absorbance of red blood cells lysed with 2% Triton X-100 was used as the positive control and taken to be 100% hemolytic, while untreated erythrocyte suspension in the PBS solution was used as a negative control. Meanwhile, a control sample of polymer solution was applied to rule out polymer absorption interference (false-positive results), and the existence false-negative results were also checked according to the reported method [32]. Each test was repeated three times. The percentage of hemolysis was calculated according to the following Equation (1):

Hemolysis (%) = [(*A*_sample_ − *A*_negative_)/(*A*_positive_ − *A*_negative_)] × 100% (1)

where *A*_sample_ represents the absorbance of the treated sample, *A*_negative_ represents the absorbance of the negative control, and *A*_positive_ represents the absorbance of the positive control.

All animal experiments in this research were carried out according to the Guidelines for the Care and Use of Laboratory Animals and were approved by the Animal Ethics Committee of Shantou University Medical College (SUMC2016-200, 5 December 2016).

### 2.7. Imaging Study of Microorganisms

Bacteria cells were grown and mixed with polymer at 2× MIC using a protocol similar to the MIC determination. After 90 min incubation at 37 °C, bacteria cells were collected, washed twice with PBS (5000× *g*, 10 min), and fixed with an equal volume of 2.5% glutaraldehyde solution for 4 h at 4 °C. Bacteria cells were rinsed in a 0.1 M PBS (5000× *g*, 10 min), and further fixed in 1% osmic acid (4 °C for 2 h). After rinsing in 0.1 M PBS, cells were dehydrated through a graded series of ethanol (30–100%).

For observation by field-emission scanning electron microscopy (FE-SEM), the dehydrated samples were transferred to a 1:1 mixture of ethanol and isoamyl acetate for 30 min, and then to absolute isoamyl acetate for 1 h. The samples were critical point-dried, sputter coated with a thin layer of gold, and analyzed with FE-SEM (SU8010; Hitachi, Tokyo, Japan).

For observation by transmission electron microscopy (TEM), the dehydrated samples were transferred to acetone for 20 min, to a 1:1 mixture of acetone and Spurr resin for 1 h, and then to a 3:1 mixture of resin and absolute acetone for 3 h. Finally, the samples were transferred to Spurr resin and incubated overnight. Sections having thickness of 70–90 nm were obtained, and post-stained with uranyl acetate and lead citrate for 10 min prior to TEM observations under a TEM setup (H-7650, Hitachi, Tokyo, Japan).

## 3. Results and Discussion

### 3.1. Synthesis and Characterization of MPLL-alt-PEG Copolymers

The PEG-crosslinked alternating copolymers MPLL-*alt*-PEG with a multi-armed PLL polyelectrolyte segment, were designed and synthesized. As demonstrated in Figure 1, PEZ was firstly synthesized through ring-opening polymerization of ZLL-NCA using PEI (Mw = 0.8 kDa) with eight peripheral primary amines as a macroinitiator [30]. Subsequently, difunctional SCM-PEG-SCM was introduced to crosslink PEZ and then the crosslinked product PEZ-*alt*-PEG was obtained under optimized crosslinking conditions [28]. The benzyloxycarbonyl protection groups of PEZ were removed in the presence of anisole and methanesulfonic acid to produce the final copolymer MPLL-*alt*-PEG. It should be mentioned that the MPLL unit in MPLL-*alt*-PEG is eight armed and the degree of polymerization of each arm is five, based on the fact that this multi-armed polypeptide was found to have broad-spectrum antibacterial activity and high selectivity from our previous study [16]. Meanwhile, MPLL-*alt*-PEG was designed and synthesized at a feeding ratio of SCM-PEG-SCM to MPLL at 1:1, since this feeding ratio is high enough for SCM-PEG-SCM to crosslink most of the MPLL unit which can also leave most of the primary amines free for the efficient electrostatic interactions between cationic MPLL segment and anionic drugs or bacterial membranes.

**Figure 2:**
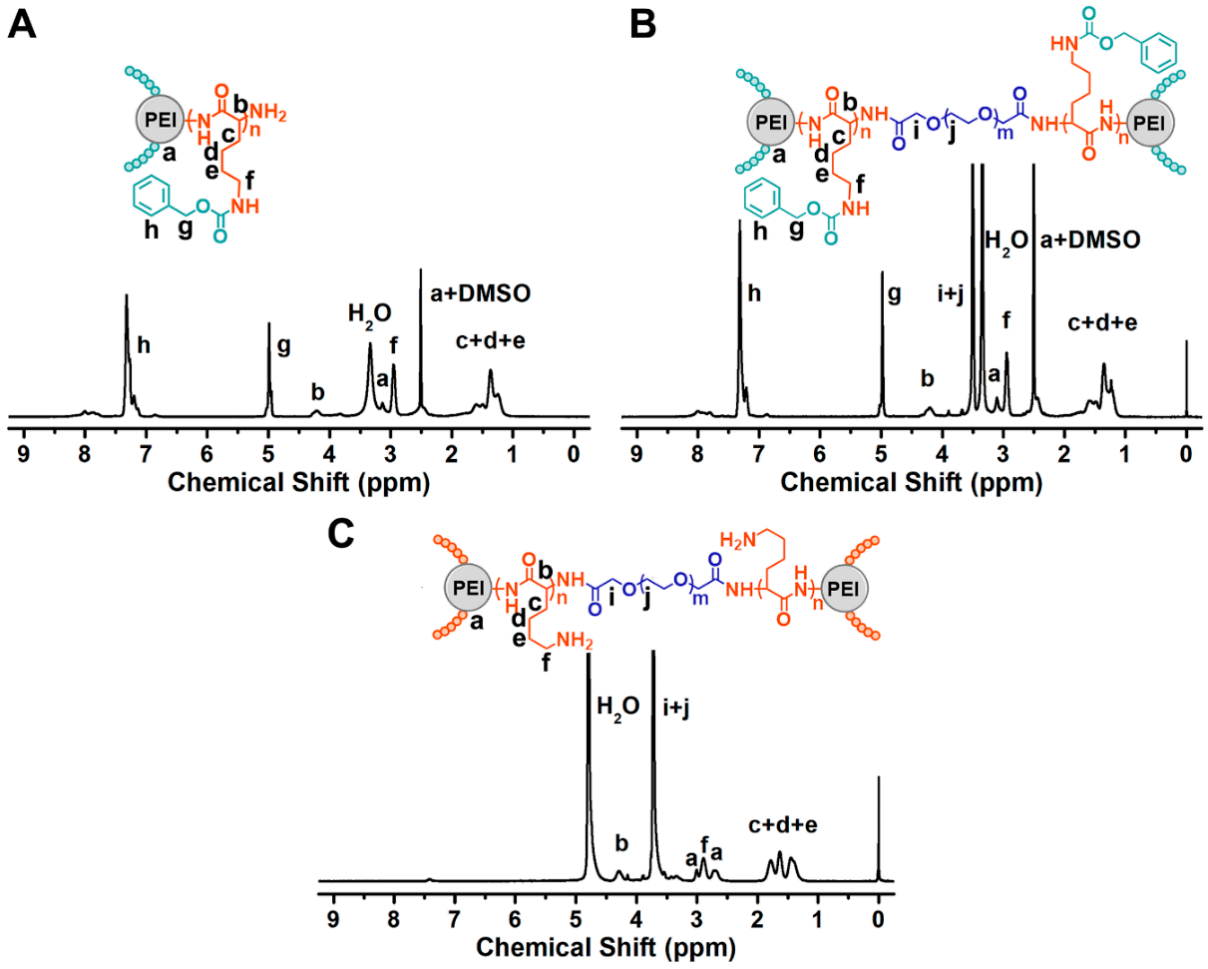
^1^H NMR spectrum of PEZ in DMSO-*d*_6_ (**A**), PEZ-*alt*-PEG in DMSO-*d*_6_ (**B**), and MPLL-*alt*-PEG in D_2_O (**C**).

Copolymers PEZ, PEZ-*alt*-PEG, and MPLL-*alt*-PEG were characterized by ^1^H NMR and the results are shown in Figure 2A–C. As shown in Figure 2B, the integration peaks at 3.4–4.0 ppm, which correspond to methylene groups (–O*CH_2_CH_2_*O–) of the PEG segment, were observed after crosslinking reaction. Using the integration peaks at 3.4–4.0 ppm of the PEG segment versus the peaks at 1.0–2.0 ppm corresponding to methylene groups of the PEZ (or MPLL) block (–*CH_2_CH_2_CH_2_*CH_2_NH–), the estimated molar ratios of PEG to PEZ (or MPLL) segments in PEZ-*alt*-PEG (or MPLL-*alt*-PEG) were found very close to their feeding ratios (Table 1). Comparing Figure 2C with 2B, the negligible residual benzyl signal at 7.3–7.5 ppm indicated that benzyloxycarbonyl groups were completely removed. These results indicated that the SCM-PEG-SCM crosslinking reaction and benzyloxycarbonyl deprotection of PEZ were proceeded in a controlled manner and MPLL-*alt*-PEG was successful synthesized.

The molecular weights of PEZ and PEZ-*alt*-PEG were measured by GPC in DMF using PMMA as the standard, while the molecular weight of MPLL-*alt*-PEG was determined in a HAc–NaAc buffer (pH of 4.5) using PEG as the standard. As shown in Table 1, the number average molecular weight (Mw) of PEZ is 6.3 kDa, while the Mw of PEZ-*alt*-PEG increased to 14.3 kDa. After the benzyloxycarbonyl protecting groups was removed, the Mw of the product was decreased to 8.5 kDa. All these results further confirmed the successful synthesis of MPLL-*alt*-PEG.

### 3.2. Preparation and Characterization of Drug-Loaded PIC Micelles

To evaluate the feasibility of MPLL-*alt*-PEG as a drug carrier, the drug-loading capacity and self-assembly of the copolymers were firstly investigated using methyl orange, an anionic water-soluble dye, as the model compound of small molecule drugs. It was expected that the primary amino groups of the MPLL segment would be protonated at pH 7.4 and carried a positive charge, thus electrostatically entrapped anionic drugs and subsequently self-assembled into PIC micelles with a MPLL/drug polyion complex core and crosslinked PEG outer shell (see illustration in Scheme 1). It revealed that MPLL-*alt*-PEG exhibited high MO loading capacity up to 52.4% ± 0.4%.

**Figure 3:**
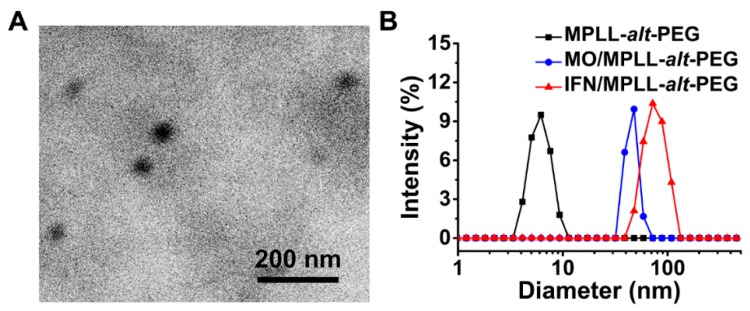
Self-assembly of MPLL-*alt*-PEG micelles. (**A**) TEM images of IFN/MPLL-*alt*-PEG micelles; (**B**) hydrodynamic diameters of free MPLL-*alt*-PEG, MO/MPLL-*alt*-PEG, and IFN/MPLL-*alt*-PEG micelles. IFN: recombinant human interferon α-2b; MO: methyl orange.

As a versatile cytokine family, type I IFNs including IFN-α and IFN-β are nowadays acting as promising therapeutic drugs with antibacterial, antiviral, antitumor, and immunomodulatory activities [33,34]. Approved by FDA for clinical treatment, type I IFNs exert definite therapeutic effects on malignant tumor, chronic hepatitis B, hepatitis C, chronic myelogenous leukemia, and other diseases [35,36]. Therefore, recombinant human interferon α-2b was chosen in our study as a model therapeutic protein for its potential in treating multifactorial diseases. In the present study, the loading capacity and encapsulation efficiency of IFN in MPLL-*alt*-PEG micelles were determined to be 16.2% ± 0.3% and 81.0% ± 0.2%, respectively. TEM measurements showed that the drug-loaded micelles adopted spherical morphologies (Figure 3A), and dynamic light scattering (DLS) measurements (Figure 3B and Table 2) indicated that the hydrodynamic diameters of MPLL-*alt*-PEG increased from 6.2 ± 1.8 to 40–80 nm upon encapsulation of MO or IFN with narrow size distributions (polydispersity index < 0.3), indicating that MO or IFN was successfully incorporated into the polymeric micelles. Free MPLL-*alt*-PEG demonstrated a positive zeta potential in a 0.01 M phosphate buffer (pH 7.4) at 25 °C, while the zeta potentials of both MO-loaded micelles and IFN-loaded micelles approached zero (Table 2), further indicating that anionic molecules were encapsulated into the polymeric micelles via electrostatic interactions.

**Figure 4:**
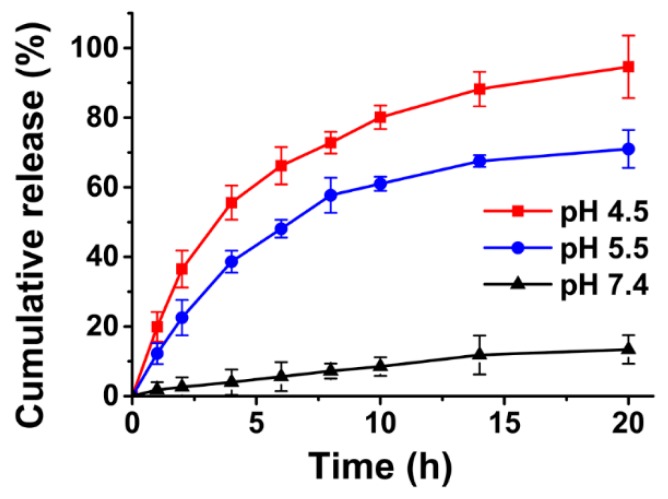
Cumulative release profiles of IFN from MPLL-*alt*-PEG at various pH.

It should be mentioned that since the isoelectric point of most proteins was in the acidic range 4–6 [37], PLL-based polymers are effective for pH-responsive protein release [24]. At physiological pH, IFN molecules (isoelectric point 5.9) carrying net multiple negative charges interact electrostatically with the cationic amino termini of the MPLL segment in MPLL-*alt*-PEG. Once IFN-loaded micelles are transferred to an acidic microenvironment, such as infectious site [38], the charging state of IFN will be changed from net negative to net positive, while the amine side-chains of MPLL remain positively charged. Therefore, the charge switching of loaded proteins may weaken the electrostatic interactions with the MPLL-*alt*-PEG host, inducing accelerated drug release. As shown in Figure 4, the release rate of IFN from the copolymer at an acidic environment was much higher than that at pH 7.4, showing a pH-sensitive release behavior. The release rate of IFN was faster at pH 4.5 than that at pH 5.5, which further indicated that the disassembly process of IFN/MPLL-*alt*-PEG micelles was dependent on the acidity of the microenvironment. The result suggested that, in the area with more bacteria and their metabolites, antimicrobial MPLL-*alt*-PEG and therapeutic proteins would be easier to be recovered for more efficient treatment.

### 3.3. In Vitro Antibacterial and Hemolytic Activities of MPLL-alt-PEG

**Figure 5:**
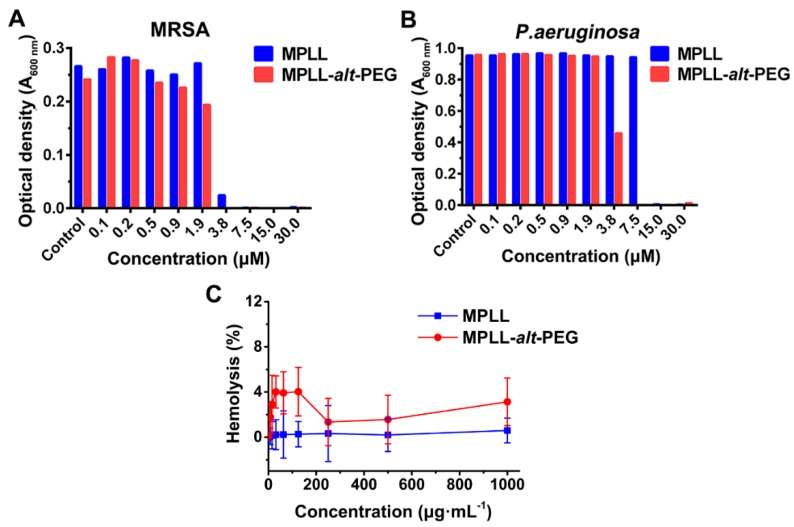
Optical density of MRSA (**A**) and *P. aeruginosa* (**B**) in MHB after incubation with various concentrations of MPLL and MPLL-*alt*-PEG copolymers for 18 h; (**C**) hemolysis of MPLL and MPLL-*alt*-PEG copolymers. MRSA: methicillin-resistant *Staphylococcus aureus*; MHB: Mueller Hinton Broth.

The antimicrobial activities of MPLL-*alt*-PEG against Gram-positive bacteria MRSA and Gram-negative bacteria *P. aeruginosa* were evaluated using MPLL as a control. MIC assays were conducted to determine the minimum drug concentration that inhibited the bacterial growth. Figure 5A,B demonstrated the optical density of MRSA and *P. aeruginosa* in MHB after the cells were treated with different concentrations of MPLL and MPLL-*alt*-PEG copolymers for 18 h. It was found that the growth inhibition of both bacterial strains was dose-dependent and the antimicrobial activity increased with increasing polymer concentration. As shown in Figure 5A,B, MPLL-*alt*-PEG showed MIC values of approximately 3.8 and 7.5 μM against MRSA and *P. aeruginosa*, respectively. In contrast, the MICs of MPLL were 7.5 and 15.0 μM, respectively. These results indicated that crosslinking the low-molecular-weight MPLL with PEG realized a higher antimicrobial activity. In this study, *P. aeruginosa* are harder to inhibit than MRSA, which may probably ascribe to the presence of an outer membrane, fewer anionic phospholipids on the membrane, and additional defense mechanisms that might be absent in Gram-positive bacteria [15,16]. It should be mentioned that, though the encapsulation of IFN may shield the amino groups of MPLL-*alt*-PEG from binding with anionic bacterial membranes, the pH-sensitive release behavior of IFN/MPLL-*alt*-PEG enable the polymer to be quickly liberated for binding bacteria at an acidic infectious microenvironment and thus exhibiting antimicrobial activity.

In addition to the high antibacterial activity, enormous selectivity for pathogens over mammal cells is also necessary to construct a potential antimicrobial agent. Since hemolysis is the mostly identified side effect of antimicrobial peptides and polymers [39], the hemolytic activity of the MPLL-based copolymers before and after PEGylation was investigated by incubating them with 5% freshly mouse red blood cells suspension at different concentrations. As shown in Figure 5C, both MPLL and MPLL-*alt*-PEG copolymers exhibited negligible hemolysis activity (less than 5%) with HC_50_ values, the concentration at which 50% of red blood cells are lysed, greater than 1000 μg·mL^−1^. The HC_50_ values of both copolymers were obviously greater than those of other natural and synthetic polypeptides [40,41], indicating a high degree of selectivity for microbes over mammal red blood cells. Since the surface of bacteria contained a large amount of anionic phospholipids, lipopolysaccharide or teichoic acids, while the outer monolayer of the mammalian membrane was composed of zwitterionic (neutral) phospholipids [29], it is speculated that the high selectively of MPLL-*alt*-PEG resulted from their preferred trend to electrostatically binding with anionic bacterial membranes.

### 3.4. Antibacterial Mechanism

**Figure 6:**
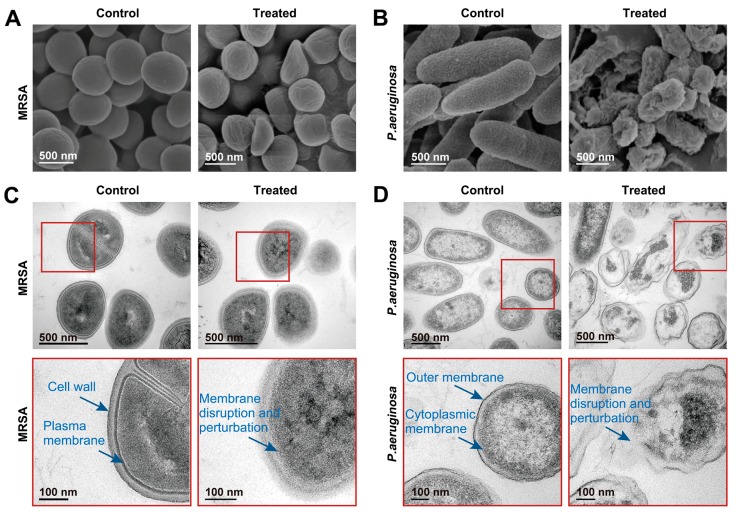
FE-SEM images of MRSA (**A**) and *P. aeruginosa* (**B**), and TEM images of MRSA (**C**) and *P. aeruginosa* (**D**) without and with MPLL-*alt*-PEG treatment.

To elucidate the antibacterial mechanism, the surface morphologies of MRSA and *P. aeruginosa* after treatment with MPLL-*alt*-PEG were investigated by visualizing the microbial cell structure using FE-SEM imaging (Figure 6A,B). Compared to the controls with smooth surfaces, microbial cells treated with cationic MPLL-*alt*-PEG exhibited distinct changes in their morphology. Wrinkled and collapsed cell walls were observed in both MRSA and *P. aeruginosa* cells treated at 2× MIC concentration. TEM images in Figure 6C,D further demonstrated the significant bacterial membrane disruption in bacteria exposed to MPLL-*alt*-PEG. As shown in Figure 6C, the plasma membrane of MPLL-*alt*-PEG-treated MRSA appears indistinct, while untreated MRSA showed intact cytoplasmic membranes. Similarly, *P. aeruginosa* cells showed intact outer membranes and cytoplasmic membranes before treatment, whereas the cell membranes of bacteria after treatment were disrupted and pores traversed the outer membrane and cytoplasmic membrane, inducing serious cytoplasmic content leakage with a large empty space in the cytosol (Figure 6D). All these results revealed that MPLL-*alt*-PEG could inhibit the growth of bacteria by a membrane-disruption mechanism similar to typical AMPs, which have been widely regarded as a promising solution to combat multidrug-resistant bacteria.

## 4. Conclusions

A new copolymer MPLL-*alt*-PEG was fabricated as both a drug carrier and cationic antimicrobial agent for the potential treatment of bacterial infection-involved multifactorial diseases. The cationic charging characteristics of MPLL segments in MPLL-*alt*-PEG could not only endow the electrostatic encapsulation of anionic drugs via the formation of PIC micelles with a MPLL/drug complex core and crosslinked PEG outer shell, but also effectively inhibit the growth of Gram-positive bacteria MRSA and Gram-negative bacteria *P. aeruginosa.* MPLL-*alt*-PEG copolymers are expected to serve as an emerging platform for combating bacterial infection-involved multifactorial diseases. The synergistic effect of antibacterial MPLL-*alt*-PEG with anionic therapeutic agents, such as anionic anti-inflammatory drug, protein, nucleic acid, and photosensitizer, has promising application potentials. The related research is currently underway and will be reported in due time.

## Figures and Tables

**Table 1:** Compositions and molecular weights of various copolymers.

Polymer	PEG:PEZ (or MPLL) in Copolymer		Molecular Weight (kDa)	
	Calc.	^1^H NMR	M_n_	PDI
PEZ	0:1	0:1	6.3	1.17
PEZ-*alt*-PEG	1:1	0.9:1	14.3	1.38
MPLL-*alt*-PEG	1:1	1.2:1	8.5	1.35

**Table 2:** Hydrodynamic diameters and zeta potential of free copolymer and drug-loaded micelles.

Samples	Diameter (nm)	Polydispersity Index	Zeta Potential (mV)
MPLL-*alt*-PEG	6.2 ± 1.8	0.237	9.2 ± 0.6
MO/MPLL-*alt*-PEG	45.7 ± 1.6	0.163	0.3 ± 0.2
IFN/MPLL-*alt*-PEG	76.8 ± 2.8	0.244	0.1 ± 0.1
